# Impact of the Acti-Pair programme on physical activity in patients with prostate cancer: protocol of the Acti-Pair 2 stepped-wedge cluster randomised trial

**DOI:** 10.1136/bmjsem-2024-002344

**Published:** 2024-12-22

**Authors:** Amandine Baudot, Evolene Fayolle, Maël Garros, Nathalie Barth, Florence Colin, Emilie Presles, Mathieu Oriol, Fanny Collange, Franck Chauvin, Aurélie Bourmaud, David Hupin

**Affiliations:** 1 U1059 INSERM, SAINBIOSE, DVH, Lyon University, Jean Monnet University, Saint-Etienne, France; 2 INSERM, CIC 1408, University Hospital Centre, Saint-Etienne, France; 3 Sport-Health House, Departmental Olympic and Sports Committee of the Loire (42), Saint-Etienne, France; 4 Auvergne Rhône-Alpes Gérontopôle, Saint-Etienne, France; 5 Presage Institute, Lyon University, Jean Monnet University, Saint-Etienne, France; 6 Clinical Research Unit, University Hospital Center, Saint-Etienne, France; 7 Support and Education Technic Centre of Health Examination Centre (CETAF), Saint-Etienne, France; 8 Clinical Epidemiology Unit, Robert Debré University Hospital, AP-HP, INSERM CIC-1426, Paris, France; 9 INSERM UMR 1137 IAME, Paris, France; 10 Department of Clinical and Exercise Physiology, University Hospital Center, Saint-Etienne, France

**Keywords:** Cancer, Physical activity, Implementation, Behaviour

## Abstract

Regular physical activity (PA) reduces morbidity and mortality in prostate cancer. Prescribing PA in cancer is a necessary awareness but is a real challenge in the case of prostate cancer. Motivational peer support seems to be an innovative strategy for lifestyle change. Therefore, we developed the Acti-Pair programme and demonstrated its feasibility. We want to evaluate its effectiveness in promoting PA in patients with prostate cancer. The Acti-Pair 2 study is an interventional, comparative, multicentre, randomised, stepped-wedge cluster study. The control group will consist of patients being followed for prostate cancer and receiving advice and recommendations for PA during consultations to make patients more active in their daily lives (=usual practice, PA to be performed independently at home). The intervention group will consist of patients being followed up for prostate cancer and benefiting from the Acti-Pair programme, which combines three interventions: (1) motivational support from a peer; (2) construction of a personalised and realistic project and (3) support from health and adapted PA professionals. This study will assess the effectiveness, implementation and efficiency of the Acti-Pair programme. It will allow the identification of key success factors for implementing the Acti-Pair programme to prepare for its transferability. Trial registration number: Clinical trial, NCT05739565, registered on 20 February 2023, https://clinicaltrials.gov/study/NCT05739565..

**WHAT IS ALREADY KNOWN ON THIS TOPIC**

- The benefits of physical activity (PA) are widely acknowledged; however, patients with prostate cancer do not engage in sufficient PA to comply with the recommendations and are not included in the programmes. Several strategies have been demonstrated to be effective in changing the behaviour of this specific population.

**WHAT THIS STUDY ADDS**

- None of the existing studies have assessed maintenance at 12 months using objective measurement nor have they combined an assessment of effectiveness, implementation and efficiency, as recommended by the Medical Research Council for complex interventions.
- The Acti-Pair programme operates through a collaborative approach involving health professionals, adapted PA professionals and peers along a clinical referral pathway, which aligns with the French policy of promoting sport and health.
- This study employs frameworks from the implementation sciences, reducing the discrepancy between research transfer and current practice.

**HOW THIS STUDY MIGHT AFFECT RESEARCH, PRACTICE OR POLICY**

- The results will provide a more comprehensive understanding of the programme’s impact on patients' with prostate cancer PA behaviour and a deeper insight into the mechanisms and processes involved in implementing the intervention and its effectiveness and efficiency. This will subsequently facilitate the transferability and scaling-up of the intervention in other contexts.

## Background

 Regular physical activity (PA), such as the 150 min of brisk walking per week recommended by the WHO, is associated with a 29% reduction in prostate cancer mortality^1^ and a 57% reduction in recurrence.^2^ Regular PA can benefit individuals with cancer by improving their physical and psychological well-being, including improving physical capacities, such as cardiorespiratory endurance and muscular strength; reducing cancer-related fatigue throughout all stages of cancer^3^; improving the overall quality of life^4^ and reducing the side effects of cancer and its treatments.^5^

However, despite the relatively favourable prognosis of prostate cancer compared with other cancers, the recognised benefits of PA in tertiary prevention, French public health policies and the sport–health facilities available in France, patients with prostate cancer reduce their PA and/or maintain high levels of sedentary behaviour after their cancer diagnosis (ONAPS, https://onaps.fr/). Physical inactivity affects 60–70% of patients.^6^ Many factors may explain these difficulties in PA adherence.

Among patients with prostate cancer receiving treatment, over 90% of those who were inactive before diagnosis remain inactive after diagnosis, and those who were previously active may become inactive following treatment with chemotherapy or hormonal therapy.^7^ The literature identifies barriers both at the individual level, including those related to treatment, disease or age-related conditions and loss of self-efficacy for PA^8^, and at the wider level, including intrapersonal, environmental and policy factors (such as limited access to PA programmes, inadequate promotion of PA by health professionals^9^ and lack of personalisation of PA programmes).^10^

Since 2017, French law modernising the healthcare system has allowed physicians to prescribe adapted physical activity (APA) for patients with long-term illnesses. A national guide and a specific reference framework to assist physicians in prescribing ‘Sport on Prescription’ to patients with cancer were provided. However, there are many barriers to physicians’ promotion and prescription of PA: lack of time, limited knowledge about PA and inadequate skills related to behaviour change.^11^ One strategy recommended in the literature for overcoming this is to provide referrals to PA experts through a PA referral system.^12^ Devices for Adapted Physical Activity Practice (DAPAPs), established in 2018 in the Auvergne Rhônes-Alpes region, are structural determinants of maintaining PA practice. DAPAP aims to strengthen the links among networks, health professionals, the public and sports associations to promote and maintain PA among individuals who are either inactive or suffer from chronic diseases.

Increasing the adherence of patients with prostate cancer to regular PA and maintaining this behavioural change over the long term is emerging as a new challenge for personalised cancer care. Strategies targeting multiple levels of ecological models (individual, intrapersonal, environmental and policy levels) are effective in improving PA behaviours.^13^ Interventions such as personalised home-based programmes,^14^ health-professional-supported PA,^15^ and peer support^16^ have already demonstrated the effectiveness of regular PA. The Acti-Pair programme, described elsewhere,^17^ targets multiple levels of PA determinants to enable these male patients to meet WHO recommendations for PA. To our knowledge, this is the first programme for patients with prostate cancer to address multiple levels of PA practice. The feasibility and viability of the Acti-Pair programme have been demonstrated in one French department; we now wish to evaluate its effectiveness on the scale of eight departments in a French region.^18^

### Study objectives

The primary aim of the Acti-Pair 2 study is to assess the effectiveness of the Acti-Pair programme on PA maintenance at 12 months compared with recommended standard clinical practice (PA advice only) in patients with prostate cancer.

The secondary objectives are to assess the impact, implementation and efficiency of the Acti-Pair programme on patients with prostate cancer.

## Methods

### Intervention: the Acti-Pair programme

**Figure 1:**
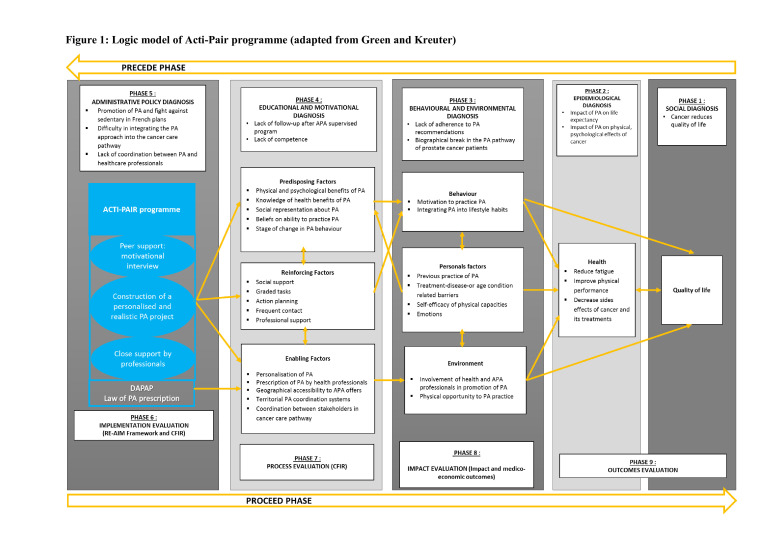
Logic model of Acti-Pair programme (adapted from Green and Kreuter). APA, adapted physical activity; CFIR, Consolidated Framework for Implementation Research; DAPAP, Device for Adapted Physical Activity Practice; PA, physical activity; RE-AIM, Reach, Effectiveness, Adoption, Implementation and Maintenance.

The Acti-Pair programme aims to initiate and maintain PA in patients with prostate cancer. The Acti-Pair programme was developed using the behaviour change wheel.^19^ Initially, we identified the barriers and levers to PA practice in physically active and physically inactive patients with prostate cancer through a qualitative analysis. We applied the Capability, Opportunity and Motivation Behaviour (COM-B) model to classify all determinants of PA and then used the theoretical domain framework^20^ to identify needed changes. Next, we determined intervention functions to identify potential courses of intervention. Third, we analysed the behaviour change techniques used in interventions found in both the literature (interventions that have demonstrated their effectiveness in increasing PA practice)^21^ and the support of DAPAP in the French region of Auvergne Rhône-Alpes. We then proceed to identify the Acti-Pair programme’s content and implementation options. Online supplemental additional file 1 presents the theoretical details of the Acti-Pair programme conception. The implementation logic model of the Acti-Pair programme is shown in figure 1.

This programme is a three-component intervention to initiate and maintain regular PA in patients with prostate cancer. (1) Motivational support from a peer (a patient with the same disease who meets the WHOs recommended level of PA), who will provide motivational follow-up, which is a social determinant to maintain PA practice over time. (2) The implementation of a personalised and realistic PA project for the patient via the DAPAP. (3) Support from healthcare professionals via the prescription of PA and from APA professional.

The details and content of the intervention are presented in table 1.

**Table 1:** Content of the Acti-Pair programme

Motivational peer support	Activities
**Training of peers** 1 day Face-to-face training in group	Skills in APA counselling techniques based on the motivational interviews (provided by a sports sociologist and a prevention and healthcare promotion professional). Knowledge of behavioural change and strategies for supporting patients at different stages of behavioural change. Knowledge of functional signs or symptoms to ensure patient safety (provided by a sports physician). Knowledge needed to determine training heart rate and perception of exertion to ensure an appropriate level of exertion (provided by an APA professional). Knowledge of existing APA devices (provided by the DAPAP coordinator). On completion of the training, peers will receive hard copies of a summary outlining the primary obstacles and motivators to PA for patients with prostate cancer who are inactive. In addition, the summary will set out strategies to overcome the barriers to PA and build on the levers. The strategies will be tailored to align with the specific behavioural change phase the patient is experiencing.
**Social support**For 12 months	**Aim:** establishing a supportive relationship with participating patients. Assessing their level of motivation. Monitoring their PA. Identifying healthcare problems and resolving barriers to regular PA. **How?** Regular communication with patients. Conducting PA sessions with patients. Offering tailored advice to help patients overcome barriers to achieving their PA goals.
**Personalised and realistic project**For 12 months	Supported by DAPAPs PA check-up. Motivational, needs and constraints assessments by APA professionals.
**Support from professionals**For 12 months	PA prescription by HP. Follow-ups at 3, 6 and 12 months by APA from DAPAPs: PA assessments, reviewing the patient’s PA project, verifying that the established objectives have been met and re-evaluating them as needed. Communication between DAPAP and HP about assessment results and all actions to implement the patient’s PA plan.

APAadapted physical activityDAPAPsDevices for Adapted Physical Activity PracticeHPhealth professionalsMETmetabolic equivalent of taskPAphysical activity

**Figure 2:**
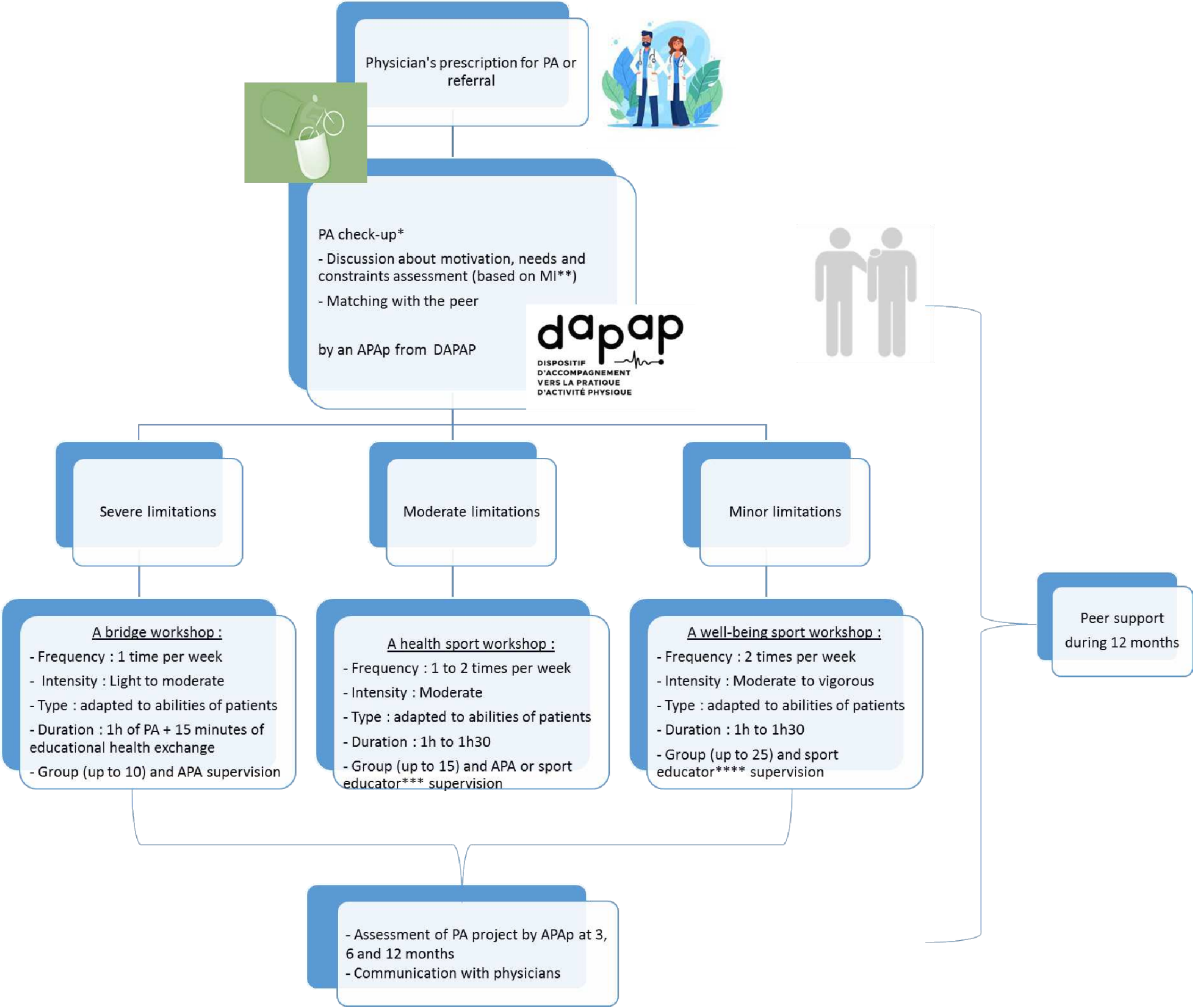
Course for patients enrolled in the Acti-Pair programme * The PA check-up will be delivered by the APAp of the DAPAP. He will assess patients' physical capacities by the 6 MWT used to assess aerobic capacity and endurance, the timed up and go test to evaluate functional mobility, leg and arm strength tests, flexibility tests and postural balance assessment. ** Motivational interviewing. *** It is expected that sports educators have completed a programme comprising 100–140 hours of training in sport and health. **** It is expected that sports educators have completed a programme comprising 3–5 days of training in sport and health. APA, adapted physical activity; APAp, APA professional; DAPAP, Device for Adapted Physical Activity Practice; PA, physical activity; 6 MWT, 6-min walk test.

The Acti-Pair programme for patients is illustrated in figure 2.

## Study design

This is a pragmatic stepped-wedge randomised controlled study of the Acti-Pair programme implementation in eight French departments, that is, eight clusters, each composed of one DAPAP, operating in the area defined by the department it is located. The intervention will be deployed in a random order, successively in the various clusters, across three stages (control, transition and interventional stages). First, all clusters start in the control and sequentially transition into the intervention (the Acti-Pair programme) until all clusters have received the intervention. Participants will be recruited successively in patients' control and intervention stages and the transition stage for peers.

**Figure 3:**
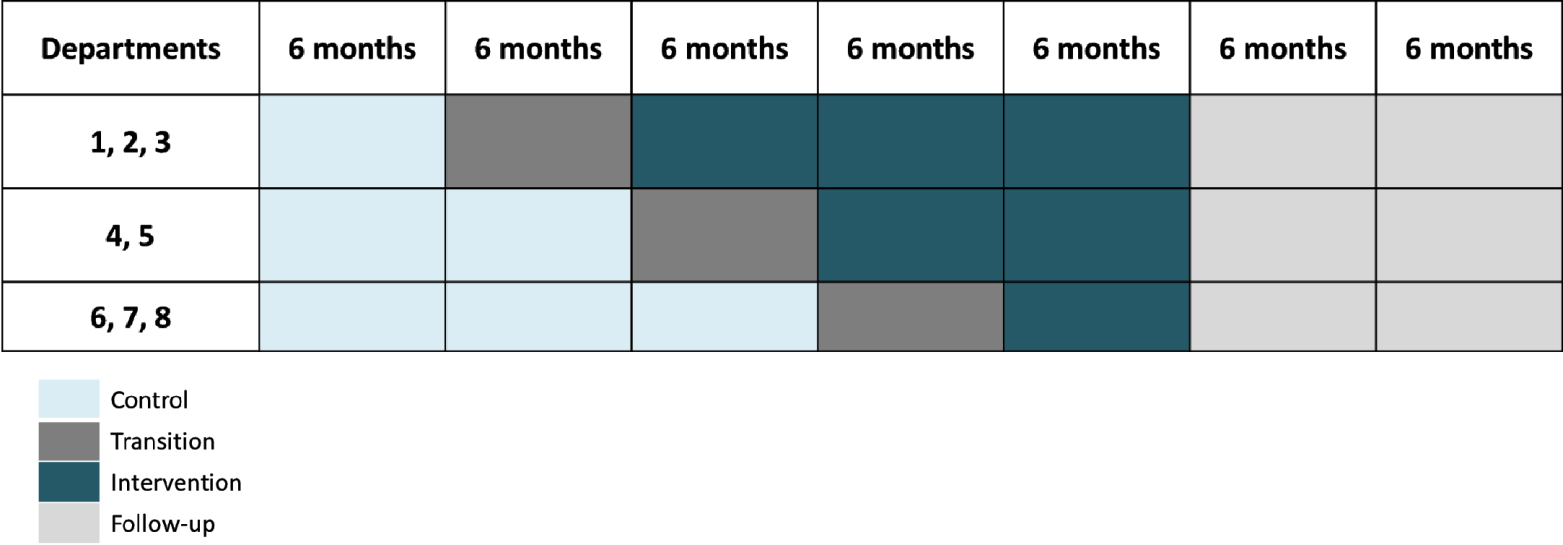
Stepped-wedge design. Each rectangle represents a cluster period.

The stepped-wedge design is presented in figure 3.

The control group will consist of patients receiving standard care for prostate cancer, which includes counselling and advice on PA during the consultation to increase daily activity levels.

The intervention group will consist of patients being treated for prostate cancer and benefiting from the Acti-Pair programme.

The stepped-wedge methodology allows for a pragmatic evaluation of the impact of a complex intervention without the limitations of individual randomisation, which focuses on individuals and does not consider the broader context in which such interventions are implemented. In addition, this methodology can be applied pragmatically and is closely adapted to the conditions of implementing the programme in the DAPAP.^22^ This methodology guarantees a high level of evidence. The sequential deployment of the intervention in the different participating DAPAPs reduces the risk of contamination that could have occurred in a parallel cluster randomised trial. It enables the potential heterogeneity of the effect of the intervention between clusters to be explored.

This protocol has been written following the standard protocol items: recommendations for interventional trials (online supplemental additional file 3) and the template for intervention description and replication (online supplemental additional file 4). The Consolidated Standards of Reporting Trials extension for the stepped-wedge cluster randomised trial has been used to optimally describe the stepped-wedge design.

### Randomisation

Each cluster will be randomly allocated to one of three sequences (two or three clusters per sequence (see figure 3)). The cluster phase allocation (Acti-Pair programme vs usual practice) will be performed by centralised randomisation via an internet platform (EnnoV Clinical software). The randomisation list will be a pseudorandomised computer list developed by a statistician before the beginning of the study. Randomisation will be stratified by cluster size. Due to the behavioural nature of the intervention, this study will not be blinded. However, the risk of bias for the primary objective assessment will be reduced because PA will be measured objectively.

### Study setting

#### Eligibility of departments

The departments will be selected based on the following.

1. The presence of a DAPAP, with an identified coordinator, which provides individual care for people (reception, advice, assessment of physical capacities and guidance towards a PA programme adapted to the health issue) with an identified coordinator.
2. The agreement of at least one specialist, per department selected, working in a hospital treating patients with prostate cancer (medical oncologist, urologist and radiotherapist).

#### Eligibility and recruitment of participants

Patients will be enrolled by hospital physicians and general practitioners who treat patients with prostate cancer by various associations in the fight against cancer and by DAPAPs. Flyers will be made available in these structures. Participants cannot participate in another trial at the same time. All eligible patients/peers, after verification of inclusion and non-inclusion criteria by the investigator, will be offered the study during a follow-up visit related to their cancer. The investigator will present the study to the patient/peer and give them the information leaflet. Written consent will be obtained from the patient/peer after a 7-day cooling-off period. The patient’s/peer’s contact details will be forwarded to the DAPAP coordinator for the department where he resides, who will contact the patient/peer.

#### Patients

Patients will be over 18 years old, diagnosed with prostate cancer for at least 1 year, practice less than 150 min of PA per week, not undergoing treatment (except hormone therapy) and have no major comorbidities preventing them from practising PA (associated cardiac pathologies, respiratory pathologies and disabling joint pathologies), have received informed information about the study by the investigator (physician or APA professional) and have cosigned a consent form to participate in the study with the investigator.

#### Peers

Peers are subject to the same eligibility criteria as patients, except for the criterion regarding PA recommendations, which requires a minimum of 150 min of PA per week.

### Outcomes

#### Primary outcome

PA levels will be assessed by an objective measurement of moderate to vigorous intensity PA (MVPA) in metabolic equivalent task (MET)-hours/week via actimetry (Actigraph GT9x, Pensacola, Florida, USA) at 12 months (±1 month) after patient inclusion in the Acti-Pair 2 study. The goal is to achieve a level of MVPA≥7.5 MET-hours/week for 1 year, that is, at least 150 min of MVPA per week, corresponding to the new WHO recommendations.

#### Impact outcomes

- PA and sedentary behaviour levels using (1) objective measures of PA (in MET-hours/week, see description of primary endpoint) and sedentary time (in h/d) via actimetry (Actigraph GT9x, Pensacola, Florida, USA) and (2) subjective measurements of PA (in MET-hours/week) and sedentary behaviour (in hours/day) using the e-Adult Physical Activity Questionnaire (EA SNA-EPIS, Université Jean Monnet, Saint-Etienne, France). This self-administered questionnaire investigated five types of PA (domestic- and work-related activities, transportation, leisure time and sports) and sedentary behaviour during the 7 previous days.
- Physical capacity by walking distance (in metres) at the 6-min walk test.
- Muscular strength by biceps muscle strength (in kilogram) on the handgrip
- Fatigue by heart rate variability, measured by the standard deviation of normal-to-normal intervals (SDNN) and root mean square successive difference (RMSSD) (milliseconds) parameters, which illustrate the general activity of the autonomic nervous system (SDNN) and the parasympathetic specific activity (RMSSD) via a heart rate monitor for 24 hours and by the Functional Assessment of Chronic Illness Therapy—Fatigue Scale questionnaire. This is a 13-question questionnaire, each corresponding to a Likert-type scale ranging from 0 to 4 (0=not at all and 4=very much). A score out of 52 is obtained. The higher the score, the less tired the patient is.
- The EuroQol-5D (EQ-5D) questionnaire will assess health-related quality of life. This is a European quality of life scale that combines five questions, each corresponding to a Likert-type scale ranging from 1 to 5 (1=no problem and 5=total incapacity) and a visual analogue scale, represented by a 20-cm line, graduated from 0 to 100, with 0 being the worst possible state and 100 being the best.

### Implementation and process evaluation

The Acti-Pair programme has the characteristics of a complex intervention because it involves diverse actors interacting in different contexts, thus offering flexibility and adaptation in its implementation.^23^ We will, therefore, evaluate the implementation of a complex intervention according to the recommendations of the British Medical Research Council published by Moore *et al*.^24^ The Reach, Effectiveness, Adoption, Implementation and Maintenance (RE-AIM) framework will be used to evaluate implementation of the programme^25^ and the Consolidated Framework for Implementation Research (CFIR)^26^ will enable us to specify the determinants that influence the implementation outcomes of the programme at different levels of stakeholders (patients, health professionals, DAPAP (managers, coordinators and APA professionals) and APA professionnals in the health and sports structure). The details of implementation and process outcomes are presented in online supplemental additional file 2.

### Health economic evaluation

A cost-utility analysis and a cost-effectiveness analysis will be performed in this study, as recommended by the French National Authority of Health (HAS, https://www.has-sante.fr/). These analyses will estimate (1) the cost per quality-adjusted life year (QALY) gained and (2) the cost per patient continuing PA at 12 months, thanks to the implementation of the intervention. The efficacy data will be those collected by the study: quality of life (using the EQ-5D-5L questionnaire) and mortality data, if any, transformed into utilities to calculate QALYs and the number of patients continuing PA at 12 months. Cost data will be collected throughout the study in the eight departments involved. The analysis will be conducted from the payer’s (health insurance) perspective. The time horizon will be the study duration (12 months), and per French guidelines (HAS, https://www.has-sante.fr/), no discount rate will be applied. The health economic analysis will include all patients participating in the study. The costs will include direct medical costs (general practitioner consultations, APA sessions, peer patients and APA professionals) and indirect costs related to the implementation of the intervention (training, rental of premises and equipment and running costs).

The outcomes and timeline are presented in table 2.

**Table 2:** Outcomes and timelines for patients, peers and professionals

	Inclusion	Control or intervention phase	3-month visit	6-month visit	12-month visit
Patients					
Consent form signature	x				
TM6	x		x	x	x
Muscular strength	x		x	x	x
FACIT-F questionnaire	x		x	x	x
APAQ questionnaire	x		x	x	x
EQ-5D questionnaire	x		x	x	x
BREQ-2 questionnaire	x		x	x	x
Actigraph GT9x, Pensacola, Florida, USA	x		x	x	x
Heart rate monitor	x		x	x	x
Medicoeconomic evaluation			x	x	x
Only for patients in the intervention phase					
WAI scale					x
Satisfaction questionnaire					x
Population reach					x
Programme adhesion					x

Peers	Inclusion	Training	End of the intervention phase
Consent form signature	X		X
6 MWT	X		X
Muscular strength	X		X
FACIT-F questionnaire	X		X
APAQ questionnaire	X		X
EQ-5D questionnaire	X		X
BREQ-2 questionnaire	X		X
Actigraph GT9x, Pensacola, Florida, USA	X		X
Heart rate monitor	X		X
Training and programme satisfaction		X	X
Adaptation: semidirective interviews	X		X
Population reach			x

Professionals	Control phase	Transition phase	Intervention phase
Adaptation and sustainability of the programme: semidirective interviews	X		X
Satisfaction questionnaire			X
Population reach			X
Medicoeconomic evaluation	X		X

APAQadult physical activity questionnaireEQ-5DEuroQol-5DFACIT-FFunctional Assessment of Chronic Illness Therapy—Fatigue Scale6 MWT6-min walk test

## Data collection

Quantitative data will be collected in each participating centre under the responsibility of each principal investigator by himself or a team member in an electronic case report form (e-CRF) (Ennov Clinical, Ennov, Paris, France). Validation checks will be automatically programmed to verify the consistency of certain data. This part will be managed by the data managers from the sponsor’s clinical research unit. At the same time, the data will be monitored by clinical research associates. Missing data, data errors or protocol violations will be specified in the data monitoring reports sent to the investigators by the sponsor.

Qualitative data will be collected through semistructured interviews conducted by a social psychologist before (control phase) and after the programme’s implementation in the departments (intervention phase). The themes addressed will be (1) evaluation of the context: factors favouring and hindering the implementation, realisation, adaptation and sustainability of the programme; compliance with the programme; (2) social representations: beliefs, knowledge and perceptions of PA, APA and the Acti-Pair programme and pathways and (3) interprofessional collaboration strategies: peers, APA professional, DAPAP coordinators and healthcare professionals.

For health economics data, physicians’ fees will be collected in addition to medical data on the e-CRF completed by physicians participating in the study and follow-up costs by peers and APA professionals via their e-CRFs. The indirect costs of training and premises rental, running costs and small purchases will be collected by the research team at the cluster level via an ‘organisational’ CRF specifically created for the cluster-level data collection. Training costs will be collected from the trainers: gross hourly wage, duration of training, travel costs, number of trainers and training sessions. The costs of premises renting and small equipment will be collected in real costs (invoices), and the follow-up carried out by the trainers will be collected the same way as for the initial training (time spent, number of trainers, salaries, number of follow-ups and travel). Costs will be valued for general practitioner’s fees at the conventional health insurance rate and sessions at unit cost (average gross salary of a peer or APA professional * time for each session * the number of sessions with the patient). Indirect costs will be valued at actual unit costs (invoices).

## Sample size

To the best of our knowledge, no study has assessed MVPA by actimetry at 12 months in patients with prostate cancer. Our hypotheses were formulated based on the results of the following. (1) Trinh *et al* have observed an initial mean level of MVPA 4.71±3.73 MET-hours/week in the exercise counselling and 6.96±7.72 MET-hours/week in the motivationally enhanced behavioural counselling group^27^ and 9.9 MET-hours/week at 3 months. (2) Livingston *et al* observed an increase of up to 12.5 MET-hours/week (>2 times the initial MVPA) at the end of the 3-month PA intervention.^15^ It is reasonable to think that a level of MVPA of between 12.5 and 7.5 MET-hours/week can be maintained at 6 months since peer support in our programme continues beyond the initial 3 months of PA. This level can be maintained at the end of 12 months of motivational support by the peer. Thus, if we estimate that we will observe the MVPA at 1 year of 5.5±4 MET-hours/week in the usual care group and 7.5±4 MET-hours/week in the Acti-Pair group, for an alpha risk of 5% and a power (1−β) of 90%, it is necessary to include 86 patients per group, that is, 172 in total. This calculation was performed using MedCalc software (MedCalc version 19.7). Assuming an intracluster correlation of 0.04 and an average cluster size of 58 participants, for 3 stepped-wedge steps, 346 patients per group will be needed, that is, 692 in total. If a drop-out rate of 10% is anticipated, a total of 762 patients will be needed.

Each peer will follow a maximum of 5 patients (Acti-Pair pilot study), and 381 patients will be followed (interventional group), that is, 77 peers. If we anticipate a drop-out rate of 10%, it will, therefore, be necessary to recruit 88 peers or 11 per department.

### Data analysis

#### Quantitative outcomes analysis

The statistical analysis will be performed on the intention-to-treat population. The statistical significance will be a two-sided alpha level of 5%. Statistical analyses will be performed using SAS software (SAS-Windows version 9.4 on computer PC) and R software version 4.2.1 on computer. The characteristics of the population will be described using quantitative variables, including the mean and SD, and qualitative variables, such as the number of cases and percentages. The Acti-Pair programme’s effect on the PA level will be analysed using a mixed-effects model with a random intercept per department to consider the correlation of the level of PA between patients of the same department and a random effect on the initial value of PA. This random effect will depend on both the cluster and the patient (random effect of the patient nested in the cluster). The model will consider the fixed effect of the treatment group (Acti-Pair programme or usual care). The possible evolution of the evaluation criterion over time will be analysed by introducing a calendar time variable into the mixed-effects regression model (5 periods of 6 months, see figure 3). If a trend is identified, the calendar time variable can be introduced into the model as an ordinal variable. This analysis will make it possible to quantify the effect of the Acti-Pair programme after adjustment for a possible time effect. An interaction between the effect of the treatment group and the random ordinal by the department may be added to quantify any heterogeneity in the benefit of the Acti-Pair programme. An initial model will be built without additional adjustment. If an imbalance between the groups is observed, a second model will be built by adjusting the variable(s) on which the imbalance is observed (sensitivity analysis). A possible logarithmic transformation of the evaluation criterion could also be considered to satisfy the normality assumption of the linear mixed-effects model.

For the analysis of the secondary endpoints, no p value will be given, and only the 95% CIs of the efficacy indices will be estimated.^28^ It will be carried out using a mixed-effects model similar to the one used for the primary endpoint. No interim analysis is planned.

#### Qualitative outcomes analysis

The analysis will be conducted using Dedoose software (version 9.0.17) by a PhD student and a social psychologist. A comparative thematic analysis of the collected content will be conducted by using the proposal by Braun and Clark, which included the following steps: familiarisation with the data, coding the data using the CFIR domains and subdomains by the two researchers, searching for themes through iterative discussion between the two researchers, comparing the themes obtained with the CFIR domains and refining the coding and illustrating with verbatims.

#### Health economic analysis

An incremental cost-utility ratio and an incremental cost-effectiveness ratio (ICER) will be calculated by dividing the difference in mean costs between the intervention group and the control group by the difference in mean effectiveness, as the French National Authority for Health (HAS) recommended. ICERs will be estimated for the two health economic endpoints, expressed as (1) the cost per QALY gained and (2) the cost per patient continuing PA at 12 months between the experimental arm and the placebo arm. These ICERs will also be produced for each department to complete the context analysis. The time horizon corresponding to the study period is 12 months; no discounting will be performed, as recommended. QALYs will be calculated based on responses to the EQ-5D-5L questionnaire, transformed into utilities and mortality data obtained during the study, if any. The efficacy endpoint for the cost-effectiveness analysis will be the number of patients who continued PA at 12 months, as measured in the study. Costs will be expressed in euros. The ICER will be positioned on the cost-effectiveness plan. Using the non-parametric bootstrap method, statistical uncertainty will be considered to construct a 95% CI and a scatter plot. A deterministic sensitivity analysis will be performed for the cost and efficiency data that appear not robust.

## Discussion

The originality of the Acti-Pair programme lies in its assessment of a long-term PA multicomponent intervention in a specific population of patients with prostate cancer affected by physical and psychological vulnerabilities, leading to social isolation and distancing from others. This population is particularly challenging in terms of behavioural change, making the Acti-Pair programme address an important issue in tertiary prevention for patients with prostate cancer.

### Acti-Pair programme: a PA referral scheme

The Acti-Pair programme allied with various healthcare professionals, DAPAPs and sports health structures to support prostate cancer remission by developing a PA programme along a referral clinical pathway. A meta-analysis has shown that the PA referral scheme process is a key motivator and driver for individuals to take up and adhere to PA interventions.^12^ Furthermore, this aligns with the French government’s objective of promoting sports and health in France by establishing sports and health centres, demonstrating the city-hospital network’s implementation.

### Real-life evaluation using implementation sciences

The Acti-Pair programme articulates several strategies for three levels of determinants (individual, intermediate and structural). Due to the Acti-Pair programme’s complex intervention nature and approval as an exercise referral scheme, a pragmatic evaluation through implementation science is required to understand the implementation process, mechanisms of action and context.^24^ This evaluation will be conducted with the help of two theoretical frameworks, RE-AIM^25^ and CFIR.^29^ The global evaluation of the impact of the Acti-Pair programme (efficacy, efficiency and implementation) would provide decision-makers with a more comprehensive insight into the elements that can aid their decision-making process.^30^ The pragmatic analysis can guide the transferability of this preventive offer to future centres. The Acti-Pair programme would serve as a foundation for a broader healthcare PA promotion programme for people with cancer or other conditions.

### Dissemination policy

The findings of this study will be disseminated in a peer-reviewed journal and presented at a scientific conference. Study participants will be duly informed of the results via mail.

## supplementary material

10.1136/bmjsem-2024-002344 online supplemental file 1

## Data Availability

No data are available.
