# Supplementary material for: Impact of the Acti-Pair programme on physical activity in patients with prostate cancer: protocol of the Acti-Pair 2 stepped-wedge cluster randomised trial
Source: BMJ Open Sport Exerc Med. 2024 Dec 22;10(4):e002344. doi: 10.1136/bmjsem-2024-002344 (PMC11664380; doi:10.1136/bmjsem-2024-002344)
Supplement: online supplemental file 1 [file bmjsem-10-4-s001.pdf]

Additional file 1: Creation of the Acti-Pair programme with the behaviour change wheel

Barriers/Levers (COM-B domains)	TDF	Intervention functions	BCTs of the Acti-Pair programme
**Physical capability:**<br>Treatment-disease-or age condition related barriers<br><br>Physical benefits of PA<br><br>**Psychological capability:**<br><br>Psychological benefits of PA	Skills<br><br>Knowledge	Training<br><br>Education	▪ Demonstration of the behaviour<br>▪ Behavioral practice / rehearsal<br>▪ Instruction on how to perform a behaviour<br>▪ Feedback on the behaviour<br>▪ Feedback on outcome(s) of behaviour<br>▪ *Behavioural practice/rehearsal*<br><br>▪ *Graded tasks*<br>▪ Information about antecedents<br>▪ Information about health consequences
**Psychological capability:**<br>PA is not a priority<br><br>Biographical disruption due to cancer	Memory, attention and decision process	Enablement	▪ Social support<br>▪ Goal setting (outcome)<br>▪ Problem solving<br>▪ Action planning<br>▪ Review behaviour goal(s)<br>▪ Review outcome goals(s)
**Physical opportunity:**<br>Personalisation of PA<br><br>Opportunity to practice PA	Environmental context and resources	Environmental restructuring	▪ *Restructuring the social environment*
**Social opportunity:**<br>Social influence<br><br>Lack of PA promotion among physicians	Social influences	Enablement	▪ Goal setting (outcome)<br>▪ Problem solving<br>▪ Action planning<br>▪ Review behaviour goal(s)<br>▪ Review outcome goals(s)<br><br>▪ Social support<br>▪ *Social comparison*<br>▪ *Demonstration of the behaviour*<br>▪ *Social reward*
**Reflective motivation:**<br>Motivation to practice PA<br><br>Sports background	Intentions<br><br>Beliefs about consequences<br><br>Goals	Persuasion<br><br>Incentivisation	▪ Salience of consequences<br><br>▪ Feedback on behaviour<br>▪ Feedback on outcome(s) of the behaviour<br>▪ Review behaviour goal(s)<br>▪ Review outcome goals(s)
**Automatic motivation:**<br>Emotions<br><br>Motivation to practice PA	Emotions	Modelling	▪ Demonstration of the behaviour<br>▪ Credible source

Legend:

BCT linked to TDF domains (in italics)

BCT integrated into the three strategies of the Acti-Pair programme: Support from health professionals, peer support, building a personalised and realistic PA project and support from PA professionals.

Note to the reader:

The barriers and levers were classified by theme and grouped according to the domains of the COM-B model. Correspondences were then established between the COM-B model and the TDF, and between the intervention functions and the BCTs.

For example, barriers related to treatment, illness or age were classified under the 'physical capacity' domain of the COM-B model. This domain is linked to the TDF competencies, which in turn are linked to the 'training' intervention function. BCTs such as 'behaviour performance/demonstration', 'behaviour practice and rehearsal' and 'instructions for performing a behaviour' are associated with the 'training' intervention function. Where a BCT is in italics, its association has been made from the corresponding domain in the TDF (and not from the intervention function). The BCT 'Behavioural practice/demonstration' is integrated into the peer support strategy (in green). The BCTs 'Behaviour practice and repetition' and 'Instructions for carrying out a behaviour' are part of the strategy for building a personalised and realistic PA project, as well as support from PA professionals (in brown).

Additional file 2: ***Details of implementation and process outcomes***

Population reach:

- *Adoption of the programme by stakeholders*: number of doctors including patients, number of peers recruited, number of peers trained, number of patients included, number of patients followed by APA professionals, number of sport-health structures involved, time dedicated by peers to the follow-up of patients, time dedicated by APA professionals to the ACTI-PAIR programme, time dedicated by health professionals to the ACTI-PAIR programme

- *Representativeness*: Adequacy of the patients included with the target population: demographic characteristics, do the oldest patients, those furthest from practice (residents in white areas, priority city districts, rural areas) participate in the programme?

- *Participation*: Number of patients, peers and professionals who agreed to participate out of the number requested, Number of sport and health facilities that participated in the programme

Adaptation:

- *Fidelity of the Acti-Pair* programme: Does each patient, peer and health professional use the programme in an adapted and planned way?

- *Adaptation of the programme*: Comparison of modifications and adaptations compared to the initial programme: training, carrying out PA assessments, organisation between different professionals

Real-life effectiveness:

- Percentage of adherence to the intervention, measured by the number of patients continuing the programme 12 months after its initiation

- Motivation to engage in physical activity for patients and peers via the behavioural regulation in exercise questionnaire (BREQ-2)(2). This questionnaire includes 6 sub-scores of motivation (introjected regulation, external regulation, intrinsic regulation, integrated regulation, amotivation and identified regulation).

- The alliance between the patient and the peer via the Working Alliance Inventory - short version (WAI-SR) (3). This questionnaire measures three dimensions: bonding (development of a positive interpersonal attachment between patient and peer including mutual trust and respect), tasks (each partner's perception of the appropriateness and effectiveness of the care tasks, as well as shared responsibility for their execution) and goals (both partners' understanding, validation and acceptance of the care change goals).

- Patients', peers', and professionals' satisfaction assessed by a questionnaire

Effectiveness of practices:

- Accessibility to health sport offers (time and geographical)

- Identification of structures offering APA

- Methods of carrying out the support: frequency, methods and duration of meetings

Sustainability of the programme:

Identification of ways to institutionalise the programme: dissemination rate, funding of the whole programme, organisation of time slots, formalisation of the process, financial facilitators, administrative recognition.
